# Pacemaker-associated Phlegmasia Cerulea Dolens Treated with Catheter-directed Thrombolysis

**DOI:** 10.5811/cpcem.2018.8.39444

**Published:** 2018-09-05

**Authors:** Benjamin A. Mazer, Patrick G. Hughes

**Affiliations:** Florida Atlantic University, Charles E. Schmidt College of Medicine, Department of Emergency Medicine, Boca Raton, Florida

## Abstract

Phlegmasia cerulea dolens (PCD) is a rare and severe form of deep venous thrombosis that is classically associated with the lower extremities. We report a case of upper extremity PCD developing abruptly in a 37-year-old female with an indwelling cardiac pacemaker who presented to the emergency department complaining of pain and paresthesias in her left arm, adjoining left chest wall, and inferior neck. Her condition was promptly diagnosed and successfully treated with intravenous unfractionated heparin and balloon venoplasty with catheter-directed thrombolysis without any known residual signs or symptoms at hospital discharge.

## INTRODUCTION

Phlegmasia cerulea dolens (PCD) is the most severe form of deep venous thrombosis (DVT) wherein the venous outflow of an extremity becomes completely obstructed.^1^ It is considered an emergency condition that can lead to venous gangrene, limb loss, and even death due to massive pulmonary embolism. This condition has an extremely high morbidity and mortality, with 20–50% of cases requiring limb amputation and 35–40% of affected patients progressing to death.^1^ PCD presents with the rapid development of a classic triad of symptoms consisting of worsening pain, swelling, and cyanosis of the affected limb.^2^,^3^ These clinical manifestations are the result of massive venous congestion that develops when the most proximal deep vein of an extremity is occluded. This leads to fluid extravasation and increased interstitial pressure, which impairs capillary blood flow.^4^ While overall uncommon, PCD of the lower extremity is well studied and characterized in the literature. PCD of the upper extremity is even more rare, with the current medical literature on the topic existing mostly in the form of individual case reports.

Unlike lower extremity DVT, which can occur spontaneously, upper extremity deep venous thrombosis (UEDVT) is typically a provoked phenomenon. UEDVT is most often associated with an indwelling foreign body such as a central venous catheter (CVC) or cardiac pacemaker/defibrillator.^5^,^6^ Herein we present a case of PCD of the left upper extremity (LUE) developing in a young female with a permanent cardiac pacemaker who had recently undergone re-implantation of a dislodged atrial lead. We discuss the factors involved in distinguishing PCD from the much more common and less-morbid symptomatic DVT and the potential benefits of educating both providers and patients about this rare yet serious complication.

## CASE REPORT

A 37-year-old female with a history of attention deficit disorder and postural orthostatic tachycardia syndrome (POTS) with neurocardiogenic syncope presented to our hospital’s emergency department (ED) complaining of an abrupt onset of pain and paresthesias in her left arm, adjoining left chest wall, and inferior neck that began one hour prior to arrival. Her surgical history was significant for transvenous cardiac pacemaker placement one year earlier, pacemaker pocket revision six weeks prior, and re-implantation of a dislodged atrial pacer lead four weeks prior. She denied history of tobacco, alcohol, or illicit drug use. She also denied any history of prior stroke, prior blood clot, or recent trauma of any kind. On review of systems, she denied any recent fevers, headache, vision change, shortness of breath, abdominal pain, nausea, or vomiting. Upon arrival, the patient’s vital signs were unremarkable. Her physical exam was significant for mildly decreased sensation to light touch over the LUE and adjoining left superior anterolateral chest wall. She had 4/5 strength in all muscle groups of the LUE with otherwise normal strength and range of motion throughout her other extremities. She had no facial asymmetry, dysarthria, mental status changes, or pronator drift. Visual inspection of her LUE revealed mild skin erythema compared to the right side. She had 2+ peripheral pulses throughout and no obvious venous engorgement.

Because of the patient’s acute onset of objective neurologic signs and concern about her unilateral dysesthesia and weakness, she received an expedited stroke workup that included laboratory studies as well as a non-contrast computed tomography (CT) of the head, perfusion CT of the head, and CT angiogram of the head and neck. Upon repeat examination when the patient was brought from the CT scanner to her room in the ED, she was noted to have developed significant edema and purple discoloration of her LUE from the shoulder to the fingers. Her LUE was cold to touch but her distal pulses were still palpable. She reported worsening pain in her entire LUE. Venous color-flow Doppler imaging had been ordered but was expedited after the findings of her repeat examination. It showed occlusive thrombus throughout the veins of the LUE including the subclavian, brachial, axillary, and basilic veins. Arterial color-flow Doppler imaging of the LUE showed normal arterial flow.

The remaining laboratory and imaging studies of her workup were negative. She was immediately started on intravenous unfractionated heparin, and vascular surgery was consulted emergently. Although the on-call vascular surgeon felt that prompt intervention was crucial, the patient’s indwelling cardiac pacemaker presented a dilemma that required input from the on-call electrophysiologist regarding the safety of removing the patient’s pacemaker. The electrophysiologist recommended against removal of the patient’s pacemaker due to the significant risks associated with her severe and debilitating POTS and neurocardiogenic syncope.

The patient was admitted to the intensive care unit, and the following morning she was taken to the operating room by vascular surgery where a venogram revealed an occlusive thrombus of the left subclavian and brachiocephalic veins extending proximally to the junction with the superior vena cava. She underwent balloon venoplasty and ultrasound-guided, catheter-directed thrombolysis using tissue plasminogen activator, after which her symptoms completely resolved. Repeat venogram after 24 hours showed near-complete resolution of the clot with some mild stenosis noted in the proximal left subclavian vein (Image). During her hospitalization she underwent a full workup for thrombophilia, which was negative. The patient was discharged on hospital day four without any residual signs or symptoms. Prior to discharge, she was started on oral rivaroxaban, which she was to continue for at least six months.

**CPC-EM Capsule**

What do we already know about this clinical entity?

Phlegmasia cerulea dolens (PCD) is the most severe form of deep venous thrombosis wherein the venous outflow of an extremity becomes completely obstructed.

What makes this presentation of disease reportable?

This young patient presented with PCD of the upper extremity associated with an indwelling cardiac pacemaker.

What is the major learning point?

Patients with an indwelling central venous catheter or cardiac pacemaker are at risk of deep venous thrombosis that can be severe and even limb- or life-threatening.

How might this improve emergency medicine practice?

Awareness of this clinical entity may allow emergency physicians to make the diagnosis and initiate therapy promptly, increasing the chance of a successful outcome.

## DISCUSSION

While UEDVT is known to be associated with indwelling devices such as CVCs and permanent cardiac pacemakers, we believe this is the first case report to detail acute venous outflow obstruction and development of PCD in a patient with an indwelling, permanent cardiac pacemaker. Nonetheless, we believe that this is an under-recognized and under-reported phenomenon that requires further attention and increased awareness. Because PCD is so strongly associated with its lower extremity variant—iliofemoral DVT—physicians are much less likely to consider it as a possibility in the upper extremity. Even in situations where a diagnosis of symptomatic UEDVT is made, physicians may not be aware that the condition is a form of PCD, with symptoms indicative of underlying microvascular (and eventually macrovascular) ischemia due to massive venous congestion.

The presenting signs and symptoms are largely dependent upon the degree of ischemia at the time. Very early in the clinical course, patients may present with more nonspecific signs and symptoms including minor arm discomfort, paresthesias, and weakness than can sometimes involve the adjacent chest wall and inferior neck.^7^ In contrast to symptomatic DVT, which tends to develop over one or several days, PCD symptoms will progress rapidly over the course of hours to include the “classic triad” of signs and symptoms including worsening pain, swelling, and cyanosis of the affected limb.^8^

Several factors likely contributed to the successful outcome of this case. First, the patient presented to the ED immediately after her initial symptoms began. This allowed emergency providers to directly observe the rapid development of the classic triad of PCD in her LUE. Additionally, the patient was very open about disclosing her medical and surgical history, including her recent procedure to revise and re-implant her cardiac pacemaker. This disclosure directed her emergency providers to consider UEDVT as a possible etiology of her symptoms.

Subclavian vein stenosis is the most common complication of permanent cardiac pacemaker implantation, occurring in some 30–50% of patients.^6^,^9^ However, this complication usually develops over a prolonged period of time, allowing for the development of collateral venous circulation. Therefore, venous thrombosis does not usually result in complete venous outflow obstruction in these individuals. There is no doubt that PCD in any setting is a rare condition. Nonetheless, in the immediate post-operative period before adequate collateral circulation develops, these patients are most prone to venous thrombosis and are at the highest risk of complete venous occlusion and PCD.^6^,^10^ From 1993–2009, the rate of pacemaker placement in the United States increased by >55%.^11^ With the increasing size of the elderly patient population in the U.S., this trend is likely to increase even more. For this reason alone, emergency physicians should become familiar with potential emergent complications of pacemaker placement, even those that are rare.

There is no firm consensus in the literature regarding the best approach to treatment of PCD. However, prompt initiation of therapeutic anticoagulation as the first step is considered standard of care. In cases of severe venous outflow obstruction with risk for progression to venous gangrene, systemic thrombolysis, surgical thrombectomy, and catheter-directed thrombolysis are available treatment options. Studies comparing the outcomes of each modality have found that catheter-directed thrombolysis is associated with the greatest reduction in risk of post-thrombotic syndrome and best chance of preventing limb loss.^3^ When successful, catheter-directed thrombolysis has the additional benefit of allowing an indwelling cardiac pacemaker to remain in place. Our case was successfully managed using this method without any known complications or adverse effects after the intervention.

## CONCLUSION

PCD is a rapidly progressive condition associated with exceptionally high morbidity and mortality. Prompt recognition of the diagnosis and initiation of therapeutic anticoagulation in the ED is paramount. Therefore, in patients presenting with upper extremity symptoms and a history of CVC, pacemaker, or defibrillator placement, emergency physicians need to consider the diagnosis of PCD. While PCD is a serious and potentially life-threatening condition, this case demonstrates that when recognized early and managed appropriately, positive outcomes are possible.

Documented patient informed consent and/or Institutional Review Board approval has been obtained and filed for publication of this case report.

## Figures and Tables

**Image:**
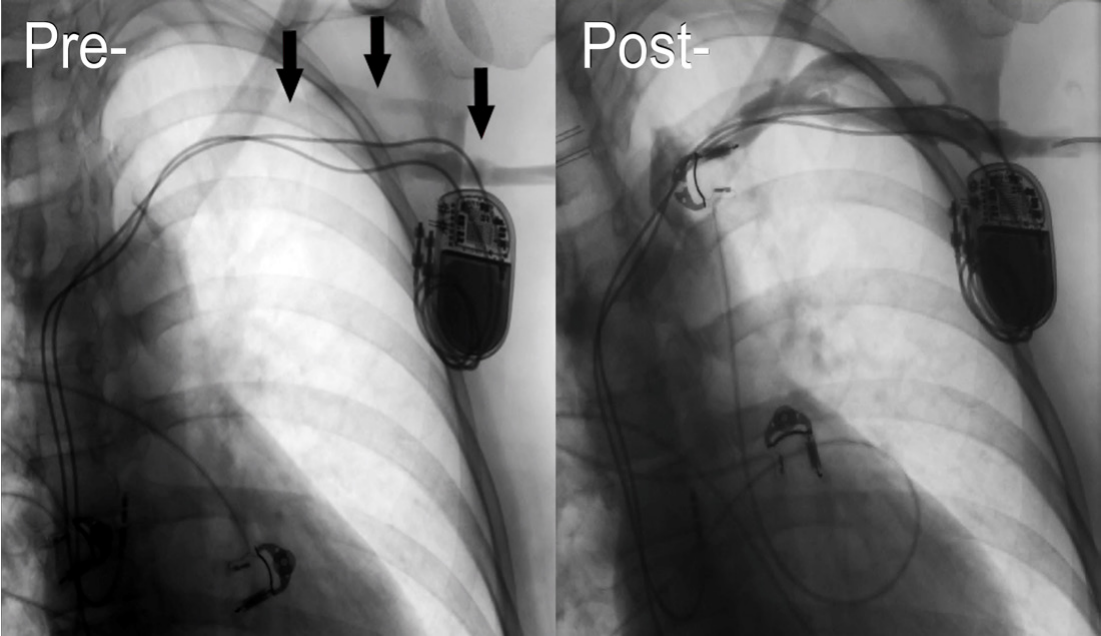
Left subclavian venogram reveals total occlusion of venous outflow from the left upper extremity on pre-venoplasty and thrombolysis imaging. Arrows indicate the site of filling defects consistent with thrombosis. Imaging obtained 24 hours post-venoplasty and thrombolysis shows marked improvement with near total resolution of the clot.
